# Neurotropism of Enterovirus D68 Isolates Is Independent of Sialic Acid and Is Not a Recently Acquired Phenotype

**DOI:** 10.1128/mBio.02370-19

**Published:** 2019-10-22

**Authors:** Amy B. Rosenfeld, Audrey L. Warren, Vincent R. Racaniello

**Affiliations:** a Department of Microbiology and Immunology, Columbia University Vagelos College of Physicians and Surgeons, New York, New York, USA

**Keywords:** acute flaccid myelitis, astrocyte, enterovirus, neuron, neurotropism, picornavirus

## Abstract

Acute flaccid myelitis (AFM) is a rare but serious illness of the nervous system, specifically affecting the gray matter of the spinal cord, motor-controlling regions of the brain, and cranial nerves. Most cases of AFM are pathogen associated, typically with poliovirus and enterovirus infections, and occur in children under the age of 6 years. Enterovirus D68 (EV-D68) was first isolated from children with pneumonia in 1962, but an association with AFM was not observed until the 2014 outbreak. Organotypic mouse brain slice cultures generated from postnatal day 1 to 10 mice and adult *ifnar* knockout mice were used to determine if neurotropism of EV-D68 is shared among virus isolates. All isolates replicated in organotypic mouse brain slice cultures, and three isolates replicated in primary murine astrocyte cultures. All four EV-D68 isolates examined caused paralysis and death in adult *ifnar* knockout mice. In contrast, no viral disease was observed after intracranial inoculation of wild-type mice. Six of the seven EV-D68 isolates, including two from 1962 and four from the 2014 outbreak, replicated in induced human neurons, and all of the isolates replicated in induced human astrocytes. Furthermore, a putative viral receptor, sialic acid, is not required for neurotropism of EV-D68, as viruses replicated within neurons and astrocytes independent of binding to sialic acid. These observations demonstrate that EV-D68 is neurotropic independent of its genetic lineage and can infect both neurons and astrocytes and that neurotropism is not a recently acquired characteristic as has been suggested. Furthermore, the results show that in mice the innate immune response is critical for restricting EV-D68 disease.

## Abstract (precis)

Since 2014, numerous outbreaks of childhood infections with enterovirus D68 (EV-D68) have occurred worldwide. Most infections are associated with flu-like symptoms, but paralysis may develop in young children. It has been suggested that infection only with recent viral isolates can cause paralysis. To address the hypothesis that EV-D68 has recently acquired neurotropism, murine organotypic brain slice cultures, induced human motor neurons and astrocytes, and mice lacking the alpha/beta interferon receptor were infected with multiple virus isolates. All EV-D68 isolates, from 1962 to the present, can infect neural cells, astrocytes, and neurons. Furthermore, our results show that sialic acid binding does not play a role in EV-D68 neuropathogenesis. The study of EV-D68 infection in organotypic brain slice cultures, induced motor neurons, and astrocytes will allow for the elucidation of the mechanism by which the virus infection causes disease.

## INTRODUCTION

The picornavirus enterovirus D68 (EV-D68) recently reemerged as an infectious cause of severe respiratory distress in young children (1–12). While the virus was originally isolated from children with pneumonia in 1962 (13), few cases of EV-D68 infection were reported (https://www.cdc.gov/non-polio-enterovirus/about/ev-d68.html) until three outbreaks in the United States and Europe in the summer and fall of 2014, 2016, and 2018 (1–10, 12, 14–16). During these epidemics, some children who were diagnosed with severe respiratory distress induced by EV-D68 infection also developed an acute flaccid myelitis (AFM) similar to that caused by poliovirus, a related enterovirus (2, 3, 6, 7, 10–12, 17). Phylogenetic analysis of the VP1 coding region of the genome suggested that EV-D68 isolates within the B1 and B3 subclades are associated with development of AFM (6, 18–22) (https://www.cdc.gov/acute-flaccid-myelitis/cases-in-us.html). However, a 1962 virus isolate not within the B clade was found to be neurovirulent in suckling mice (13). Apart from two clinical samples, EV-D68 has not been isolated from the cerebrospinal fluid or blood of infected children with AFM (23, 24).

EV-D68 is a unique enterovirus, as its physical and genetic properties are similar to both human rhinoviruses (HRVs) and poliovirus. Akin to the HRV particle, the EV-D68 virion is acid labile and optimal virus growth occurs at 33°C (13, 25, 26). Like HRVs, the sites of infection for EV-D68 are the nasopharyngeal cavity and the respiratory tract, but not the oropharyngeal and intestinal mucosa, which are sites of entry for poliovirus. Transmission of EV-D68 is also very different from that of poliovirus and occurs via respiratory aerosols, not fecal-oral contamination (27). Yet, EV-D68 disease is extremely different from that caused by either HRVs or polioviruses as infected patients do not develop a viremia. Unlike poliovirus, EV-D68 is rarely shed in the feces of infected patients (27, 28), because infectivity is destroyed by the low pH of the stomach (25).

Children with AFM associated with EV-D68 display limb weakness and cranial nerve abnormalities (6, 17, 18, 29, 30). It has been suggested that the ability of the virus to infect neural cells (astrocytes and neurons), or neurotropism, is a recently acquired phenotype (6, 31–34). This conclusion has been made by using neuroblastoma cells, which are not primary cells of the nervous system. Two murine models of EV-D68-induced paralytic myelitis have been established. These two models address the question of neuroinvasion, the ability of the virus to enter the central nervous system (CNS) from a peripheral site of infection, and neurovirulence, the ability to cause disease. One model utilizes infection of 1-day-old mice, and intramuscular inoculation of the virus is required for efficient development of paralysis (35). Unlike poliovirus, EV-D68 is a respiratory virus not known to infect the musculoskeletal system, nor has virus been observed to traffic into the CNS via the neuromuscular junction. Furthermore, the respiratory tract is a passive conduit for gas exchange and, with the exception of the diaphragm, lacks skeletal muscle. In this model, virus replication in the brain was not detected. However, radiological images of EV-D68-infected patients have identified lesions within the brain stem, specifically the cranial nerve motor nuclei (18, 30–32, 36, 37). In a second model, mice lacking the alpha/beta/gamma interferon (IFN-α/β/γ) response inoculated intraperitoneally or intranasally develop limb paralysis (38, 39), yet evidence of viral replication is absent.

We used multiple approaches to determine if EV-D68 isolates from 1962 to 2014 are neurotropic, by determining whether they replicate within astrocytes and neurons, using murine organotypic brain slice cultures, which allow for the separation of neurotropism and neuroinvasion; primary murine astrocytes; human induced neurons and astrocytes; and *ifnar* knockout mice. As sialic acid binding varies among these isolates, it was also possible to assess the role of this sugar in neurotropism.

## RESULTS

### EV-D68 infection of mouse neuroblastoma and embryonic fibroblasts.

**FIG 1:**
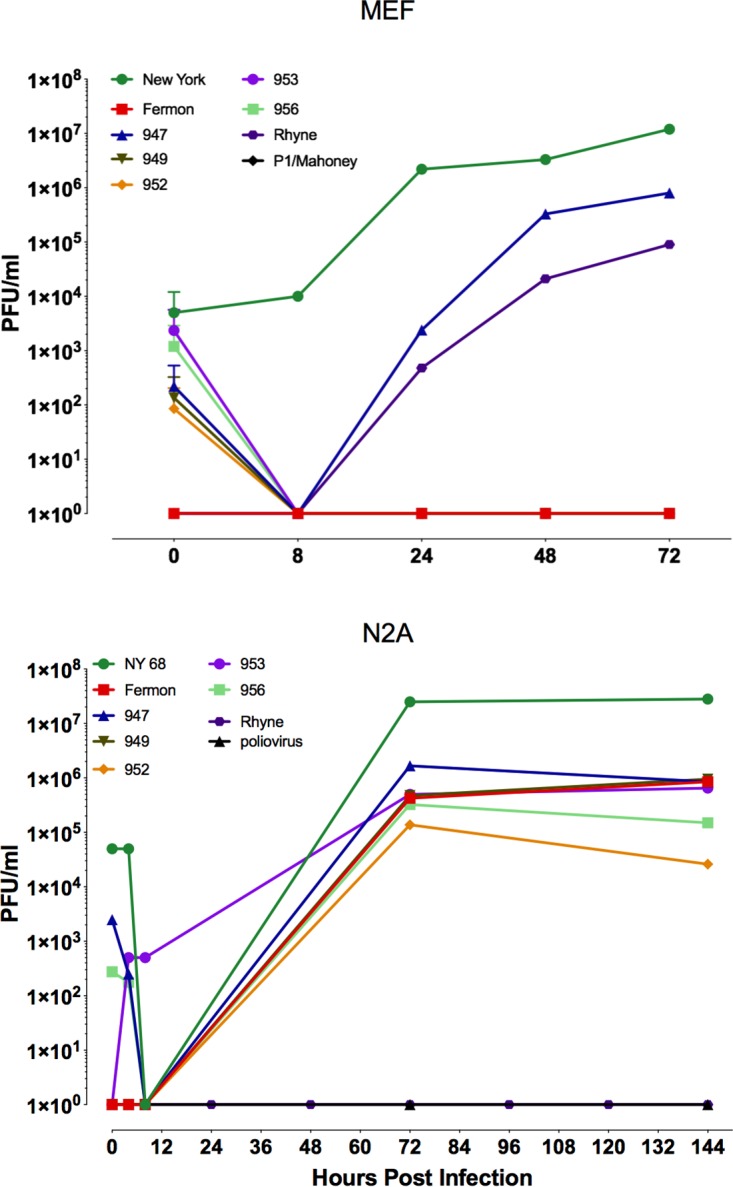
Replication of EV-D68 isolates in mouse embryonic fibroblasts and neuroblastoma cells. Time course of replication of multiple isolates of EV-D68 from a mouse embryonic fibroblast (MEF) cell line and a mouse neuroblastoma cell line (N2A). Cells were infected with virus at a multiplicity of infection of 3 and incubated at 33°C, and at the indicated times, cultures were harvested and assayed for infectious virus by plaque assay. Results are representative of three independent experiments.

To determine if mouse cells are susceptible and permissive for EV-D68 infection, a mouse embryonic fibroblast cell line (MEF) and a mouse neuroblastoma cell line (N2A) were infected with multiple isolates of EV-D68 including Fermon and Rhyne from the initial virus outbreak in 1962; NY from 2009; and 2014 isolates, including 947, 949, 952, 953, and 956. Virus replication was assessed by plaque assay. The NY, 947, and Rhyne isolates replicated in MEFs, while all but the Rhyne isolate replicated in the N2A cultures (Fig. 1). Poliovirus 1/Mahoney was used as a negative control; both murine cell lines lack human CD155, the cell receptor for poliovirus. These results demonstrate that some but not all isolates of EV-D68 can replicate in mouse cells.

### Interaction of EV-D68 and sialic acid on the surface of cells.

Several reports, including the initial description of the virus, have shown that, *in vitro*, sialic acid binds the virus capsid (13, 40–43). Furthermore, the crystal structure of sialic acid bound to EV-D68 revealed that sialic acid binds within the canyon located at the 5-fold axis of symmetry (41, 44). To determine if sialic acid interacts with multiple isolates, hemagglutination assays were performed using red blood cells (RBCs) from both guinea pigs and humans. Nine different batches of human RBCs comprising 3 donors per batch were used. Fermon and three isolates from 2014 (947, 949, and 956) agglutinated guinea pig RBCs; no isolate agglutinated human RBCs (Table 1). The absence of human RBC agglutination was previously observed using EV-D68 isolates from the 2012 outbreak in the Netherlands (40).

**TABLE 1:** Hemagglutination of EV-D68 isolates on guinea pig and human red blood cells

Virus isolate	Hemagglutination titer	
	Human	Guinea pig
New York	0	0
Fermon	0	256
Rhyne	0	0
947	0	256
949	0	64
952	0	0
953	0	0
956	0	512
Influenza virus A/WSN	256	1,024

### EV-D68 neuraminidase resistance is cell type and isolate specific.

**FIG 2:**
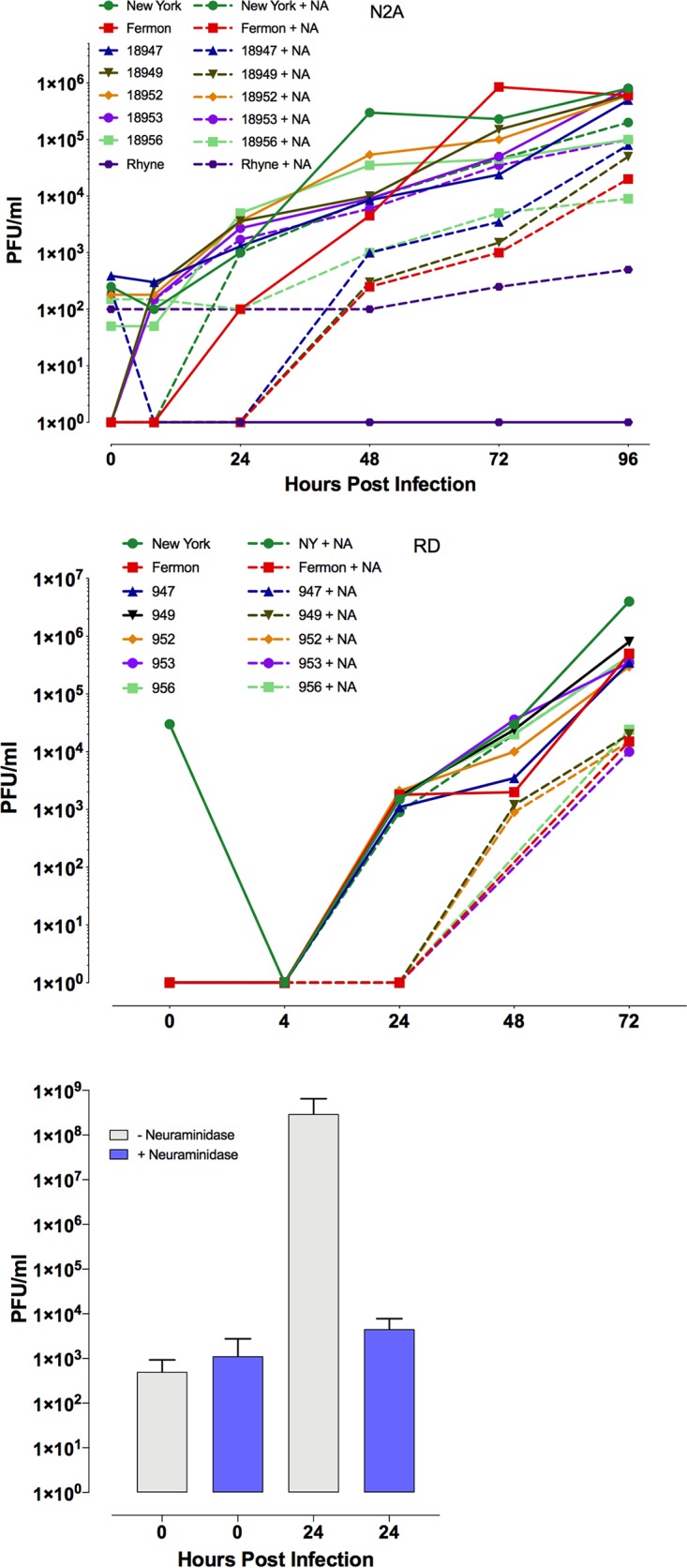
Effect of neuraminidase on replication of EV-D68 isolates. Time course of replication of multiple isolates of EV-D68 from RD and N2A cells untreated or treated with neuraminidase at 37°C for 30 min prior to virus infection. Cells were infected with virus at a multiplicity of infection of 3 and incubated at 33°C, and at indicated times, cultures were harvested and assayed for infectious virus by plaque assay. Results are representative of three independent experiments. Bottom, effect of neuraminidase treatment on yield of influenza virus. MDCK cells were pretreated with neuraminidase as described above, infected with influenza virus at a multiplicity of infection of 3, and incubated at 33°C. At 24 h postinfection, supernatants were harvested and assayed for infectious virus by plaque assay.

The requirement for sialic acid can also be investigated by its removal from the cell surface with neuraminidase prior to virus infection. To further refine understanding of the requirement for sialic acid for infection with EV-D68, cultures of various cells, including rhabdomyosarcoma (RD) cells, the cells used for propagation and titration of the virus, and N2A cells, were incubated with medium containing neuraminidase 30 min prior to infection to remove sialic acid. At the indicated time postinfection, viral replication was determined by plaque assay. All EV-D68 isolates with the exception of Rhyne were able to infect neuraminidase-treated N2A cells, albeit with diminished efficiency (Fig. 2). The effect of neuraminidase treatment on viral yield varied depending on the isolate and between cell types. The Rhyne isolate did not replicate in N2A cells; however, upon neuraminidase treatment, a higher background titer was observed, although no replication took place (Fig. 2). Neuraminidase treatment might have prevented efficient removal of nonabsorbed virus despite two washings with phosphate-buffered saline (PBS) after absorption. This observation may reflect a role for sialic acid in increasing the local concentration or proper localization of the cell receptor within the cell membrane; infection with the Rhyne isolate might be more dependent upon these properties. Alternatively, the Rhyne isolate might have a lower affinity for the cell receptor than other isolates, and the presence of sialic acid aids could increase the affinity/avidity of the interaction between virion and receptor; consequently, entry into neuraminidase-treated cells is slower for this isolate than others, leading to a higher background after adsorption. Without knowledge of the receptor and receptor-virus particle kinetics, it is difficult to resolve this observation. The activity of neuraminidase was assessed by incubating Madin-Darby canine kidney cells or human RBCs with neuraminidase-containing medium for 30 min prior to the addition of influenza A virus. Production of infectious virus after infection of Madin-Darby canine kidney cells pretreated with neuraminidase was severely attenuated (Fig. 2). Additionally, no hemagglutination by influenza A virus was observed when human RBCs were pretreated with neuraminidase (data not shown). These results suggest that interaction with sialic acid may be necessary in only certain contexts to facilitate virus attachment to the cell surface, as described for Theiler’s murine encephalomyelitis virus, enterovirus 70, and coxsackievirus A24 (45–49).

### Replication of EV-D68 in induced human neurons and astrocytes.

**FIG 3:**
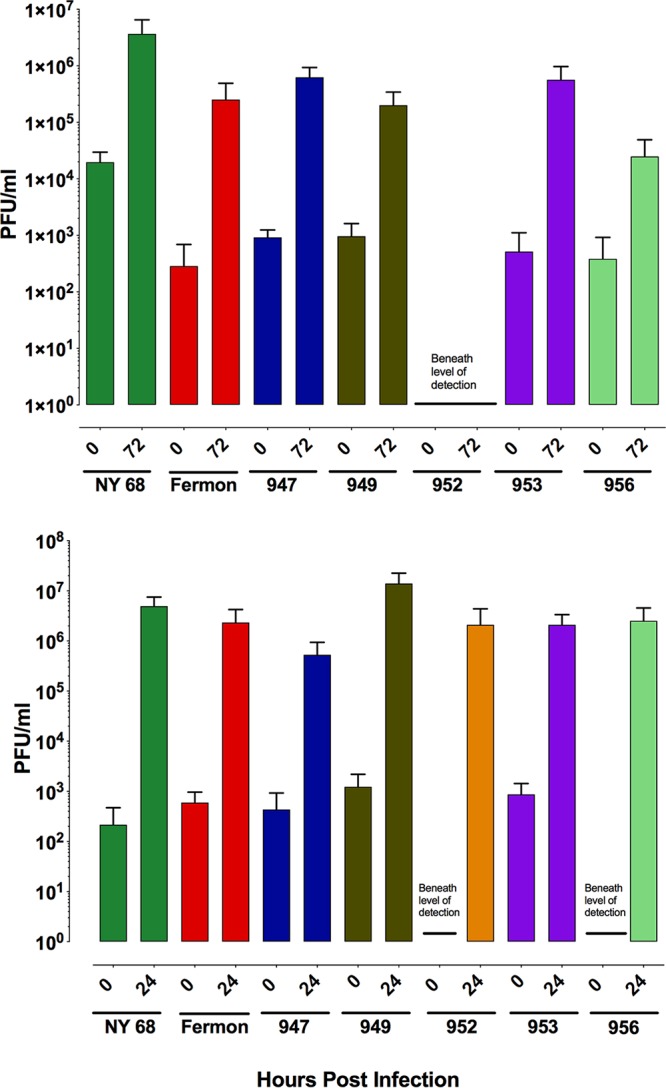
Replication of EV-D68 isolates in human cortical neurons derived from induced pluripotent stem cells. Time course of replication of multiple isolates of EV-D68 from hiPSCs cortical neurons or astrocytes derived from induced human pluripotent stem cells. Cells were infected with virus at a multiplicity of infection of 3 and incubated at 33°C, and at indicated times, cultures were harvested and assayed for infectious virus by plaque assay. Results are representative of three independent experiments.

To determine if EV-D68 can productively infect cells of the human CNS, cortical neuron and astrocyte cultures derived from human induced pluripotent stem cells (hiPSCs) were infected with multiple virus isolates. Sensory, inhibitory, and excitatory neurons along with motor neurons can be found within cortical neuron cultures. Culture medium was collected at various times postinfection, and virus production was assessed by plaque assay. Replication of all isolates except 952 was observed in cultures of induced neurons (Fig. 3). All seven isolates of EV-D68 replicated in human induced astrocyte cultures (Fig. 3).

### Replication of EV-D68 in postnatal murine organotypic brain slice cultures.

Organotypic brain slice cultures have been widely used to define genes involved in neurological development and defining disease mechanisms (50, 51) and for initial testing of viral vectors for gene therapy (52). Results from such studies have complemented findings from existing mouse models. We used murine brain slice cultures to demonstrate that neurotropism is not a recently acquired phenotype of Zika virus (53).

**FIG 4:**
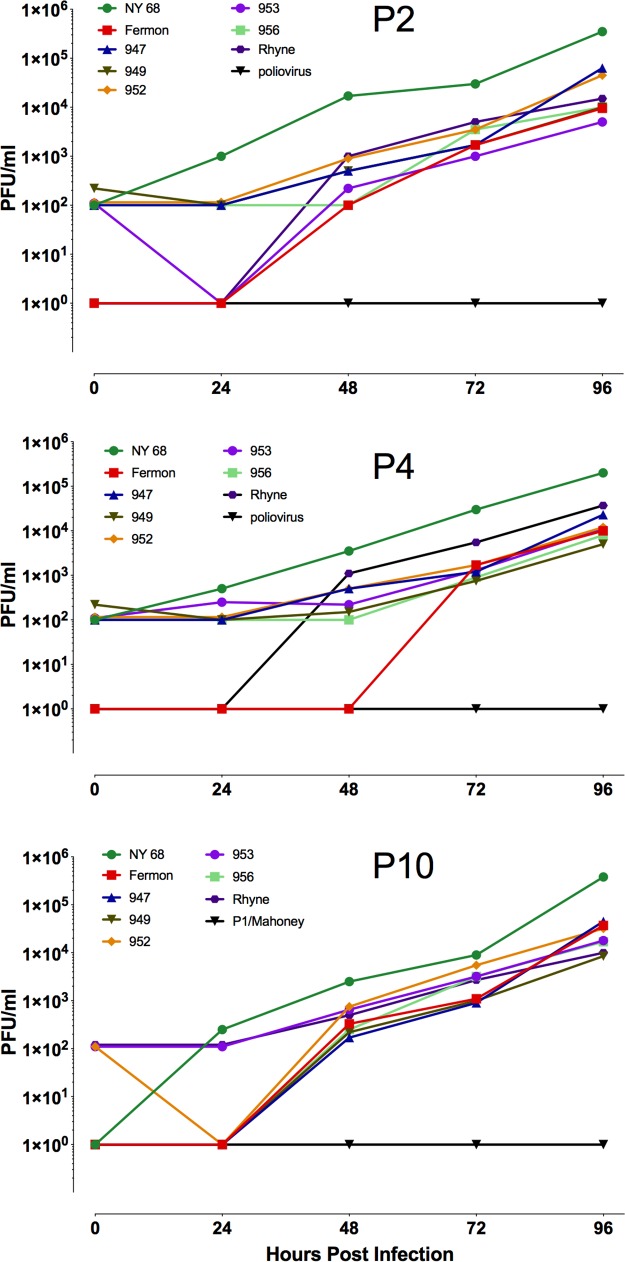
Replication of EV-D68 isolates in organotypic brain slice cultures generated from postnatal day 2, 4, and 10 mice. Time course of replication of multiple isolates of EV-D68 in organotypic brain slice cultures produced from postnatal day 2 (P2), 4 (P4), and 10 (P10) mice. Cultures were infected with 10^5^ PFU of virus and incubated at 33°C. At indicated times, cultures were harvested and assayed for infectious virus by plaque assay. Results are representative of three independent experiments.

**FIG 5:**
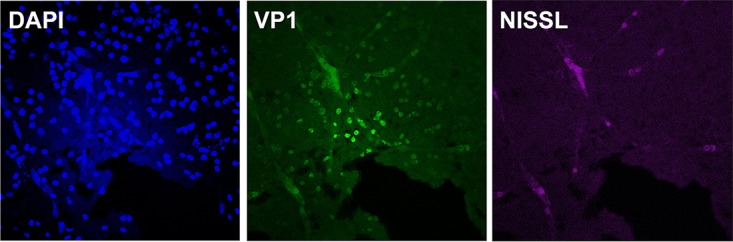
Indirect immunofluorescence microscopy of EV-D68-infected organotypic brain slice cultures. Indirect immunofluorescence microscopy of organotypic brain slice cultures generated from postnatal 4-day-old mice infected with EV-D68 947 isolates. At indicated times, cultures were fixed and stained with antibody to DAPI (blue), anti-EV-D68 VP1 antibody (green), and Nissl stain (purple).

**FIG 6:**
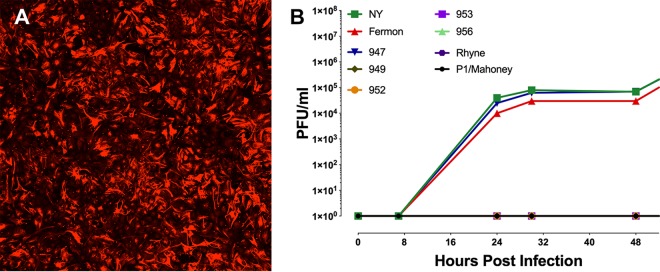
Replication of EV-D68 isolates in primary murine astrocytes. (A) Primary murine astrocytes stained with antibody against GFAP. (B) Time course of replication of multiple isolates of EV-D68 in astrocytes isolated from P1 to P3 mouse brains. Cells were infected with virus at a multiplicity of infection of 3 and incubated at 33°C, and at indicated times, cultures were harvested and assayed for infectious virus by plaque assay. Results are representative of three independent experiments.

The effect of EV-D68 infection on organotypic brain slices was determined using cultures generated from P2, P4, and P10 mice. Virus replication was assessed by plaque assay at various times postinfection. All EV-D68 isolates productively infected brain slice cultures from P2, P4, and P10 mice (Fig. 4). As expected, poliovirus did not replicate in murine brain slice cultures. Indirect immunofluorescence microscopy using a monoclonal antibody that specifically recognizes VP1 of EV-D68, as well as NeuroTrace Nissl stain, a dye that selectively binds Nissl substance found within in the cytoplasm of neurons within the brain and spinal cord, revealed that all EV-D68 isolates infected Nissl-stained neurons (Fig. 5 and data not shown). Viral antigen was also observed in cells that did not stain with Nissl, possibly astrocytes (Fig. 5). To determine if murine astrocytes can be infected with EV-D68, glial fibrillary acidic protein (GFAP)-positive astrocytes were isolated from brains of P1 and P3 postnatal mice and infected with all isolates. Three of seven EV-D68 isolates (NY, Fermon, and 947) productively infected these cells (Fig. 6).

### EV-D68 infection of mice lacking the type I interferon response.

**FIG 7:**
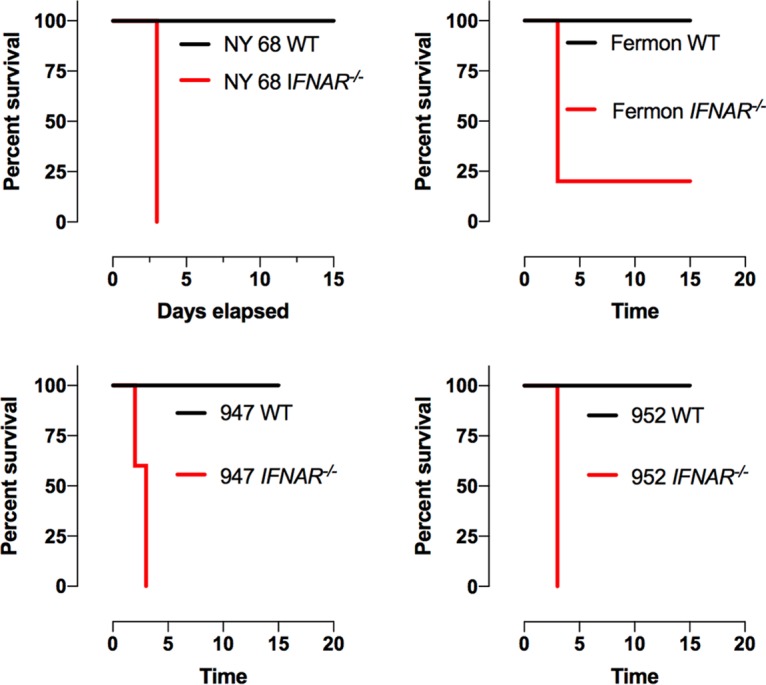
Survival plots of mice infected with EV-D68. Four-week-old wild-type and *ifnar*^−/−^ mice were infected with 10^5^ PFU of either the NY 68, Fermon, 947, or 952 isolate of EV-D68. No wild-type mice developed paralysis. All but one *ifnar*^−/−^ mouse developed paralysis 3 days postinfection.

EV-D68-associated paralysis is predominantly diagnosed in children under the age of 6 years. To determine if neuropathogenesis of EV-D68 infection of mice is age dependent, 10-day- and 4-week-old wild-type C57/BL6 animals were intracranially inoculated with 10^5^ PFU of the NY 68, Fermon, 947, and 952 isolates. No mouse developed paralysis (Fig. 7 and data not shown). The host innate immune response is thought to restrict tissue tropism of other picornaviruses (54–58). To determine if the innate immune response restricts EV-D68 neuropathogenesis, 4-week-old mice (9 females and 11 males) lacking the interferon α/β receptor (*ifnar*^−/−^) were intracranially inoculated with 10^5^ PFU of the NY 68, Fermon, 947, and 952 isolates. By 3 days postinfection, all but one mouse developed paralysis, which was independent of sex (Fig. 7). These data suggest that EV-D68 infection of the central nervous system is restricted by the innate immune response.

## DISCUSSION

Four mouse models of EV-D68 infection have been established (13, 35, 59–61). One model examined the immune response within the respiratory tract elicited after intranasal inoculation with EV-D68 using 8- to 10-week-old wild-type BALB/c mice; however, no virus replication was observed in these animals (61). The other three models use either postnatal day 1 or immunocompromised mice to examine neuroinvasion and neurovirulence of EV-D68. Paralysis was observed after intracranial or intramuscular inoculation with multiple isolates of EV-D68 in 1-day-old mice (35). In-depth characterization of virus replication and induction of paralysis, however, was determined for only one EV-D68 isolate, and approximately 50% of the animals were paralyzed after intracranial inoculation. However, development of paralysis following intracranial inoculation of suckling mice with the Rhyne isolate was first reported in 1967 when EV-D68 was initially described (13). These data question the conclusion that neurotropism and neurovirulence are a recently acquired phenotypes of EV-D68 infection. Furthermore, little to no neuronal dysfunction was observed when 1-day-old mice were intranasally infected, which is the natural route of infection in humans. Moreover, in this same mouse model paralysis occurred most frequently when mice were intramuscularly injected (35). Yet, virus replication within skeletal muscle was not assessed. It remains unknown if EV-D68 can infect and replicate within skeletal muscle and if the production of infectious progeny correlates with the onset of paralysis, as observed in the murine model of poliomyelitis (62). Despite the absence of evidence that EV-D68 replicates in skeletal muscle, the authors suggest that EV-D68 may invade the CNS in a manner similar to poliovirus—via the neuromuscular junction—but this hypothesis was not addressed, e.g., by severing the nerve that innervates the limb prior to intramuscular inoculation to impair trafficking. EV-D68 is a respiratory virus not known to infect the musculoskeletal system in patients; rarely is a viremia established during human infection, and aside from the diaphragm, the respiratory system lacks skeletal muscle. The mechanism by which the virus traffics from the respiratory tract into the CNS is not known. Moreover, postnatal day 1 mice are known to develop paralysis when intracranially injected with a Sabin vaccine strain of poliovirus (63), questioning the utility of using 1-day-old mice as models for EV-D68 pathogenesis.

The second mouse model of EV-D68-associated neurologic dysfunction examines the outcome of infection in adult mice either intracranially or intranasally inoculated with the virus. These animals are immunocompromised, lacking the IFN-α, -β, and -γ genes. Results from these mouse studies as well as our results reported here indicate that EV-D68 infection is sensitive to the type I and II interferon responses. These observations reinforce the role of the interferon response in limiting picornavirus infection (54–58) but are inadequate for the establishment of an animal model that can be used to investigate how subtleties in host genetics influence disease development and progression, to determine whether neurological dysfunction directly results from virus replication or immune infiltration into the CNS, or to facilitate the development of novel antiviral therapeutics and vaccines against virus infection (64).

Other animal models of EV-D68 infection, including cotton rats and ferrets, have also been described. These models have been predominantly used to investigate the respiratory disease associated with virus infection. In the cotton rat model, replication in the respiratory tract and the induction of neutralizing antibodies were studied after intranasal inoculation with the virus (59). In this model, no obvious pathology within the respiratory tract was observed in virus-infected animals, which is in contrast to the result observed in EV-D68-infected air-liquid interface cultures of human bronchial epithelium, nor was virus replication observed (65). A transient viremia developed in ferrets after intranasal inoculation, and virus-specific RNA was detected in feces from infected animals, neither of which is observed during most human infections. No neurological disease developed, nor did virus replicate in this animal model of EV-D68 infection (60).

Organotypic brain slice cultures are ideal for separating neurotropism from neuroinvasion and for an in-depth study of EV-D68 neurotropism. Neuroinvasion, the ability to enter the CNS from the periphery, may not only reflect how the subtleties of host genetics modulate the host-pathogen interaction but may also reflect differences between isolates dictated by the biochemical and biophysical properties of the virion. Moreover, sensitivity to the innate immune response may vary between isolates, influencing the neuroinvasive and neurovirulence properties of the virus. Although production of brain slices severs nerve connections, and the cultures lack immune cells from the periphery that infiltrate the CNS during virus infection, organotypic brain slice cultures nevertheless maintain many aspects of *in vivo* brain biology, including functional local synaptic circuitry with preserved brain architecture and vascularization and the presence of environmental cues required for neuronal function, and can remain viable for several days in culture. Their utility has been well established in hundreds of publications. They have been extensively used to understand neuronal connectivity and neurodegenerative disorders and are the experimental system for initial testing of viral vectors for gene therapy (52, 66–69). They have also been used to study Zika virus and measles virus infections (53, 70, 71). Findings from organotypic brain slice cultures complement those from established animal models and can be used to elucidate questions of mechanism and cell biology. Organotypic brain slice cultures allow for the direct assessment of neurotropism of a virus by placing the virus inoculum directly on target cells. Intracranial inoculation does not fully separate neuroinvasion from neurotropism, because virus is also delivered to the space surrounding the brain. Organoid cultures, while very popular, are unable to faithfully mimic the cellular and structural complexity of the postnatal brain, are heterogeneous with respect to cell type composition and structure, become hypoxic as they increase in size, and lack external cues which influence the reproducibility of any findings (72).

The results of phylogenetic analysis of EV-D68 isolates suggest that infection with viruses from specific evolutionary lineages (subclades B1 and B3) is associated with the development of AFM (6, 31–34). This postulate has been furthered by work examining EV-D68 infection of both undifferentiated and differentiated human neuroblastoma-derived SH-SY5Y cells. These cells were initially defined to be “neuronal like” by the presence of dopamine-β-hydroxylase activity and more recently by comparison of their transcriptome with that of HeLa cells, an ovarian cancer-derived cell line, not with that of neurons (34). Despite being morphologically similar to neurons, these cells were initially isolated from the bone marrow of a patient with neuroblastoma and are known to lose their neuronal characteristics upon passage. Consequently, SH-SY5Y cells are less suitable for defining viral neurotropism than neurons induced from stem cells or organotypic brain slice cultures.

To understand EV-D68 neurotropism, we examined isolates from multiple lineages, including subclade B1, initially associated with the outbreak of AFM in 2014, for the ability to infect the mouse neuroblastoma cell line N2A; organotypic brain slice cultures from day 1 to 10 postnatal mice, developmental stages that correlate with the years 1 to 6 in human brain development; primary murine astrocytes and human neurons and astrocytes derived from induced pluripotent stem cells; and mice lacking the IFN-α/β receptor. We find that the EV-D68 isolates examined can replicate in N2A cells, brain slice cultures, and 4-week-old mice lacking the IFN-α/β receptor. The exception is the Rhyne isolate, which did not replicate in murine N2A cells. Nevertheless, this isolate did replicate in organotypic brain slice cultures derived from wild-type mice. The Lansing isolate of poliovirus type 2 displays a similar phenotype, in that it replicates in and causes paralysis in wild-type mice but yet does not replicate in mouse cell cultures (73). The basis for this phenotype is not yet understood.

Based on the observations made in organotypic brain slice cultures, we conclude that EV-D68 is neurotropic independently of its genetic lineage and that neurotropism is not a recently acquired phenotype as has been suggested elsewhere (6, 31–34, 74). Instead, our data align with recent phylogenetic analysis demonstrating that EV-D68 sequences from AFM patients do not form a monophyletic group but are interspersed among the clades (20, 35). Furthermore, the observation that 6% of 1-day-old mice intracranially inoculated with the Rhyne isolate of EV-D68 did develop paralysis adds additional support for our hypothesis that neurotropism of the virus is not a newly acquired phenotype (35). It is possible that recent changes in the genome of EV-D68 allow invasion of the virus into the central nervous system from the periphery. This hypothesis seems unlikely because a major selective force for viral evolution is transmission to a new host. Once a virus enters the central nervous system, it cannot be transmitted to another host. Therefore, unless the evolution of neurovirulence was an accidental consequence of other selective forces, selection for neuroinvasion seems highly unlikely.

An important question is why EV-D68 did not induce paralysis after intracranial inoculation of virus into 10-day-old wild-type mice, but replication was observed in organotypic brain slice cultures derived from mice of the same age. One possible explanation for these observations is that paralysis is immune mediated, dependent upon immune cells which infiltrate the CNS from the periphery. This hypothesis is consistent with the observation that children infected with EV-D68 develop paralytic disease 8 to 10 days after diagnosis of the respiratory disease.

We find that all EV-D68 isolates replicate in human iPSC-derived cortical neurons, except for isolate 952 (Fig. 5), which replicates, together with all other isolates, in human iPSC-derived astrocytes. However, the 952 isolate was most efficient at inducing limb weakness in the 1-day-old murine model of EV-D68 pathogenesis and was recently found to infect human iPSC-derived motor neurons (35, 74). Differences in cells or virus stocks might explain why we did not observe replication of this isolate in human iPSC-derived cortical neurons, in contrast to the findings from others in mice. Only 3 of the 7 EV-D68 isolates productively infected primary murine astrocytes. The observed difference between human iPSC-derived astrocytes and primary murine astrocyte cultures may be explained by the methods for preparation of the cultures. Induced astrocytes are clonal, generated by reprogramming a population of cells, while primary murine astrocytes are composed of terminally differentiated positionally defined cells. The regional position of astrocytes influences induction of the interferon response; the cerebellar astrocytes possess a more efficient antiviral response than those found in the cerebral cortex (75). The observation that only a subset of EV-D68 isolates replicated in primary astrocyte cultures may reflect differences in the sensitivity of isolates to the interferon response.

In contrast to the infection of human neurons and astrocytes by other isolates, the virus titer at time zero was below detection for the 952 and 956 isolates. As the virus titer after the adsorption period is influenced by receptor-mediated uncoating of the particle, this observation suggests that there are relevant differences in capsid structure and acid sensitivity among these isolates. This hypothesis is consistent with reported differences in uncoating of EV-D68 isolates and rhinoviruses by low pH (25).

Presumably, replication in neurons would lead to the symptoms of AFM, including muscle and cranial nerve dysfunction. Despite astrocytes being the major producer of IFN within the CNS, the mechanism by which virus replication in astrocytes would lead to neuronal dysfunction and paralysis is not known. Replication of EV 71 in astrocytes is thought to participate in development of paralysis observed in virus-infected humans, nonhuman primates, and mice (76). A role for infection of astrocytes has also been proposed to be part of the neuropathology caused by the flaviviruses West Nile virus and Zika virus and the alphavirus chikungunya virus (75, 77–79). The ability of West Nile virus to infect astrocytes not only is a distinguishing characteristic of pathogenic and nonpathogenic isolates but leads to the release of neurotoxic factors responsible for neuronal apoptosis (80, 81). A role for astrocytosis in motor neuron destruction during amyotrophic lateral sclerosis has also been proposed (82).

The identity of the receptors for EV-D68 remains poorly defined. ICAM-5 has been suggested to function as a proteinaceous receptor for EV-D68 in the brain (43). However, production of ICAM-5 is restricted to the telencephalon, the most rostral segment of the brain region; consequently, it is not present on the surface of cells within the brain stem or spinal cord, two sites where lesions are found in radiological images of EV-D68-infected children (18, 30–32, 36, 37, 74, 83). Furthermore, ICAM-5 is not found on the surface of epithelial cells that line the respiratory tract, the primary site of EV-D68 infection (http://www.proteinatlas.org/ENSG00000105376-ICAM5/tissue). The cells that we use to propagate EV-D68, including RD cells, N2A cells, MEFs, and induced human neurons and astrocytes, do not produce cell surface ICAM-5 as determined by flow cytometry, nor do they produce ICAM-5 mRNA (data not shown). The absence of ICAM-5 on the surface of induced human neurons has also been confirmed by others (74). A recent genome-wide CRISPR/Cas9 screen to identify cell genes whose encoded proteins are needed for the early steps of viral infection failed to retrieve ICAM-5 (84).

The role of sialic acid in EV-D68 infection is also unclear. Certain isolates of the virus can hemagglutinate red blood cells and can replicate in cells treated with neuraminidase to remove sialic acid (13, 40, 42, 44). This sugar binds the viral capsid on the eastern wall of the canyon located at the 5-fold axis of symmetry, displacing a C_14_ fatty acid from a hydrophobic pocket, allowing for the partial collapse of the canyon (41, 44). However, VP4 density was still observed in the structure of the EV-D68–sialic acid complex. Moreover, no change in the size of the virion was observed in the presence of sialylated receptor analogues at room temperature (data not shown), suggesting that sialic acid may only initiate the first step of the entry process, attachment to the cell surface, and is not involved in conformational changes related to uncoating (44). Inactivation of the gene encoding CMP-*N*-acetylneuraminic acid synthase in cells, which is required for synthesis of sialic acids, impairs replication of some, but not all, isolates of EV-D68 (40). However, it was not demonstrated that lack of sialic acid impairs virus binding to cells. Disruption of sialic acid production not only affects protein glycosylation but also impairs synthesis of glycolipids. The formation and function of intracellular vesicles and proper fusion between these vesicles are dependent upon glycolipids. Such vesicles are critical for the entry and RNA synthesis of enteroviruses. Our results confirm that removal of sialic acid from the cell surface with neuraminidase impairs infection with specific isolates of EV-D68, but it is important to note that such treatment only allows for the function of extracellular sialic acid to be assessed. We also find that the ability of EV-D68 to hemagglutinate guinea pig red blood cells varies according to isolate, and no isolate that we examined hemagglutinated human red blood cells. Nevertheless, all of the EV-D68 isolates replicate in organotypic brain slices from mice. We conclude that binding to sialic acid is not essential for infection of the brain. These data have been confirmed by others (74).

The results presented here establish organotypic brain slice cultures as a pharmacologically and genetically amenable system to study EV-D68 neurotropism and elucidate the mechanism of virus-induced damage, with distinct advantages over live animals and organoid cultures. For example, when assessing the effects of experimental drugs on viral replication in brain slice cultures, the added compounds are confined to the culture medium, rather than potentially altering the physiology of the entire animal. Slice cultures can be produced from mice of any genetic background, and DNA for mutant alleles can be readily introduced by *ex vivo* electroporation prior to generating cultures to study the effects of host protein variants on viral replication. The alternative, to knock in alleles in mice, is far more time- and reagent-consuming. The use of brain slice cultures, induced motor neurons, and astrocytes will provide mechanistic insight into EV-D68 neurotropism.

## MATERIALS AND METHODS

### Ethics statement.

All experiments were performed in accordance with guidelines from the *Guide for the Care and Use of Laboratory Animals* of the NIH (85). Protocols were reviewed and approved by the Institutional Animal Care and Use Committee (IACUC) at Columbia University School of Medicine (assurance number AC-AAAR5408).

### Cells, mice, and organotypic brain slice cultures from neonatal mice.

Rhabdomyosarcoma (RD) cells were grown in Dulbecco’s modified Eagle’s medium (DMEM; Invitrogen, Carlsbad, CA), 10% fetal calf serum (HyClone, Logan, UT), and 1% penicillin-streptomycin (Invitrogen). Human induced cortical neurons and astrocytes were purchased from Cellular Dynamics, Fujifilm, and grown according to the manufacturer’s specifications (Cellular Dynamics, Madison, WI).

C57/BL6 and *ifnar*^−/−^ mice were bred in a specific-pathogen-free facility at Columbia University Medical Center.

At the appropriate time postbirth, C57/BL6 mice were euthanized per animal study protocol and brains from were dissected into ice-cold artificial cerebrospinal fluid (ACSF) consisting of 125 mM NaCl, 5 mM KCl, 1.25 mM NaH_2_PO_4_, 1 mM MgSO_4_, 2 mM CaCl_2_, 25 mM NaHCO_3_, and 20 mM glucose, pH 7.4, 310 mosmol^−1^. Brains were embedded in 4% low-melting-point agarose dissolved in ACSF and sliced into 300-μm coronal sections using a vibratome (Zeiss). Slices were maintained on 0.4-μm, 30-mm-diameter Millicell-CM inserts (Millipore) in cortical culture medium (CCM) containing 25% Hanks balanced salt solution, 47% neurabasal MEM, 25% normal horse serum, 1× penicillin-streptomycin-glutamine, and 30% glucose. Cultures were maintained in a humidified incubator at 37°C with constant 5% CO_2_ supply.

### Viruses.

Enterovirus D68 isolates (EV-D68) US/MO/14-18947 (947), US/MO/14-18949 (949), US/IL-14-18952 (952), US/KY/14-18953 (953), and US/IL/14-18956 (956) were obtained from BEI Resources. The numbers in parentheses are used to refer to each isolate in the text. Rhyne and New York (NY) EV-D68 isolates were kindly provided by Shigeo Yagi (California Department of Public Health, Richmond, CA) and W. Ian Lipkin (Mailman School of Public Health, Columbia University), respectively. The Fermon isolate was purchased from ATCC (American Tissue Culture Collection; Manassas, VA). All viruses were propagated and assayed in RD cells. Viral titers were determined by plaque assay (see below).

### Binding assays.

Hemagglutination assays were done in V-shaped microtiter plates (Fisher, 07-200-698). Equal volumes of 0.5% washed human (David Fidock, Columbia University, NY) or guinea pig (Rockland, R402-0050) red blood cells were added to serial 2-fold dilutions of virus. Plates were incubated at 4°C, and readings were made every 30 min for 2 h. Activity of neuraminidase was verified by lack of influenza virus hemagglutination on treated guinea pig red blood cells (data not shown).

### Indirect immunofluorescence microscopy.

At either 0 h or 96 h postinfection, the medium was removed from EV-D68 virus-infected or uninfected organotypic brain slice cultures and the cultures were placed overnight in 4% paraformaldehyde (PFA) fixative dissolved in 1× PBS at 4°C. Following fixation, cultures were incubated in blocking solution of PBS, 0.3% Triton X-100, and 3% horse serum. Cultures were incubated overnight in at 4°C in blocking solution containing appropriate primary antibodies. Sections were washed in 1× PBS and incubated in the presence of fluorophore-conjugated secondary antibody in blocking solution. Sections were mounted on slides using Aqua-Poly/Mount (Polysciences, Inc.) or Fluorsave (Millipore, Fisher) and imaged using an iZ80 laser scanning confocal microscope (Olympus FV100 spectral confocal system). Brain sections were imaged using an 60× 1.42-numerical-aperture (NA) oil objective or a 10× 0.40-NA air objective. All images were analyzed using ImageJ software (NIH, Bethesda, MD, USA).

### Infection of *ifnar*^−/−^ mice.

Mice were inoculated intracerebrally with 10^5^ PFU of EV-D68 using a 1-ml tuberculin syringe. Mice 10 days old were inoculated with 15 μl, and mice 4 weeks old were inoculated with 50 μl. Five mice were inoculated with each virus isolate or PBS. Mice were observed daily for paralysis or death, and paralyzed mice were sacrificed immediately.

### Plaque assay.

RD cells were seeded on 60-mm plates for approximately 70% confluence at the time of plaquing. Next, 100-μl portions of serial 10-fold virus dilutions were incubated with cells for 1 h at 37°C. Two overlays were added to the infected cells. The first overlay consisted of 2 ml of DMEM, 0.8% Noble agar, 40 mM MgCl_2_, and 10% bovine calf serum. After solidification, a second liquid overlay was added that was composed of 1 DMEM, 0.1% bovine serum albumin, 40 mM MgCl_2_, 0.2% glucose, 2 mM pyruvate, 4 mM glutamine, and 4 mM oxaloacetic acid. The cells were incubated at 37°C for 4 to 6 days and developed by using 10% trichloroacetic acid and crystal violet.

### Virus infections.

Organotypic brain slice cultures were infected with 10^5^ PFU of EV-D68 isolate NY, Fermon, Rhyne, 947, 949, 952, 953, or 956 or poliovirus type 1. Virus was allowed to adsorb to the slices for 1 h at 37°C. The inoculum was removed, and the slices were washed twice in 1× PBS. Infected slices were cultured in CCM for 96 h.

### Antibodies.

Antibodies used in this study were rabbit polyclonal antibody against VP1 of enterovirus D68 (Genetex, 132134, 1:250 dilution), rabbit polyclonal antibody against glial fibrillary acidic protein (GFAP) (Abcam, ab53554), and rabbit polyclonal allograft inflammatory factor (Iba1) (Wako, 019-19741). Donkey fluorophore-conjugated secondary antibodies (Jackson Laboratory, 1:500 dilution) were used together with DAPI (4′,6-diamidino-2-phenylindole; Thermo Scientific, 62248, 1:1,000 dilution) and NeuroTrace Nissl stain (Life Technologies, N21483).

### Data analysis.

GraphPad Prism software was used to analyze all data. Log_10_-transformed titers were used for graphing the results of plaque assays.
